# Cardinal features of involuntary force variability can arise from the closed-loop control of viscoelastic afferented muscles

**DOI:** 10.1371/journal.pcbi.1005884

**Published:** 2018-01-08

**Authors:** Akira Nagamori, Christopher M. Laine, Francisco J. Valero-Cuevas

**Affiliations:** 1 Division of Biokinesiology and Physical Therapy, University of Southern California, Los Angeles, California, United States of America; 2 Department of Biomedical Engineering, University of Southern California, Los Angeles, California, United States of America; Johns Hopkins University, UNITED STATES

## Abstract

Involuntary force variability below 15 Hz arises from, and is influenced by, many factors including descending neural drive, proprioceptive feedback, and mechanical properties of muscles and tendons. However, their potential interactions that give rise to the well-structured spectrum of involuntary force variability are not well understood due to a lack of experimental techniques. Here, we investigated the generation, modulation, and interactions among different sources of force variability using a physiologically-grounded closed-loop simulation of an afferented muscle model. The closed-loop simulation included a musculotendon model, muscle spindle, Golgi tendon organ (GTO), and a tracking controller which enabled target-guided force tracking. We demonstrate that closed-loop control of an afferented musculotendon suffices to replicate and explain surprisingly many cardinal features of involuntary force variability. Specifically, we present 1) a potential origin of low-frequency force variability associated with co-modulation of motor unit firing rates (i.e.,‘common drive’), 2) an in-depth characterization of how proprioceptive feedback pathways suffice to generate 5-12 Hz physiological tremor, and 3) evidence that modulation of those feedback pathways (i.e., presynaptic inhibition of Ia and Ib afferents, and spindle sensitivity via fusimotor drive) influence the full spectrum of force variability. These results highlight the previously underestimated importance of closed-loop neuromechanical interactions in explaining involuntary force variability during voluntary ‘isometric’ force control. Furthermore, these results provide the basis for a unifying theory that relates spinal circuitry to various manifestations of altered involuntary force variability in fatigue, aging and neurological disease.

## Author summary

Involuntary fluctuations in muscle force are an unavoidable consequence of human motor control and underlie movement execution errors. Amplification and distortion of involuntary force variability are common phenomena found in various neurological conditions and in fatigue. However, the underlying mechanisms for this are often unclear. We investigated the generation and modulation of involuntary force variability arising from different sources, as well as their interactions. We used a closed-loop simulation which included a physiologically-grounded model of an afferented musculotendon and an error-controller. We show that interactions among neural noise, musculotendon mechanics, proprioceptive feedback, and error correction are critical components of force control, and by taking these into account, our model was able to both replicate and explain many cardinal features of involuntary force variability previously reported experimentally. Also, our results suggest previously unrecognized pathways through which force variability may be altered in fatigue and in certain neurological diseases. Finally, we emphasize the potential for important clinical and scientific information to be extracted from relatively simple, non-invasive measurements of force.

## Introduction

Involuntary fluctuations in muscle force are inherent to human motor control. Evidence suggests that this apparent ‘noise’ is functionally significant for movement execution and learning [1–5]. Furthermore, amplification of force variability or distortion of its frequency content is an almost universal phenomenon whenever neuromuscular control is altered, for example by aging [1, 6], fatigue [7, 8], and neurological diseases [9–13]. However, whether such phenomenon is caused by common or distinct factors is not known because the sources of involuntary force variability and their potential interactions are not well understood.

By some descriptions, involuntary force variability is a manifestation of broad-band neural noise [3–5]. However, neural drive to muscles is known to have a highly structured frequency spectrum [14]. Accordingly, different neural sources of involuntary force variability, such as descending drive [15–18] and proprioceptive feedback [19–22], are often described specifically in terms of their frequency content. Frequency-specific force variability can also stem from mechanical sources (e.g. mechanical resonance), even if the neural drive itself contains no distinct oscillatory components [23, 24]. Attempts to understand the relative contribution that each ‘source’ of involuntary force variability makes to the total have been difficult, given that they all act concurrently during muscle activation, and are difficult to experimentally isolate and manipulate.

While different sources of involuntary force variability may be distinct, they are not likely to be independent. For example, there is recent evidence suggesting an inverse relationship between low (1-5 Hz)- and high-frequency (5-12 Hz) neural drive to muscles [25–27]. The high-frequency drive may originate from stretch-reflex circuitry [19, 22]. The low-frequency drive (the so-called ‘common drive’) does not have a known origin, but appears to be negatively influenced by Ia afferent feedback, since it is strongest in muscles which have low spindle densities [25]. Further, experimental conditions which increase high-frequency neural drive and H-reflex amplitudes also decrease low frequency neural drive [26, 27]. Together, the clear implication is that Ia afferent feedback oppositely affects high and low frequency neural drive (and thus force variability), but the mechanistic details are not yet understood.

In this study, we establish how neural and mechanical sources of force variability interact to produce the structured force spectrum observed experimentally using a physiologically-grounded model of afferented muscle. Our simulation of an afferented musculotendon set inside of a closed-loop control scheme allowed us probe the mechanistic interactions that exist among an error correction mechanism for muscle force, proprioceptive feedback, and mechanical properties of muscle. Further, we describe these interactions in terms of their effects on involuntary force variability and on the behavior of a simulated pool of motor units.

Our hypotheses were 1) neuromechanical interactions inherent to the closed-loop control of viscoelastic musculotendon would suffice to produce low-frequency force variability, 2) tuning of proprioceptive feedback (i.e., known modulation of fusimotor drive or presynaptic gains) would impact the entire frequency spectrum of force variability, and 3) those changes in force variability would be reflected in motor unit synchronization.

Our findings not only support these predictions, but (i) emphasize the importance of neuromechanical interactions to levels not previously recognized, and (ii) they describe how isolated changes in each proprioceptive pathway gain influences the full spectrum of involuntary force variability. This novel demonstration fills a critical gap in our understanding of how error correction mechanisms, proprioceptive feedback, noise, and musculotendon mechanics are interrelated, and our results emphasize the critical importance of investigating involuntary force variability within the context of closed-loop control. Our results are an important step towards a unifying theory that relates spinal circuitry to various manifestations of altered involuntary force variability in functional performance [1], aging [1, 6], fatigue [7, 8] and neurological disease [9–13].

## Results

**Fig 1:**
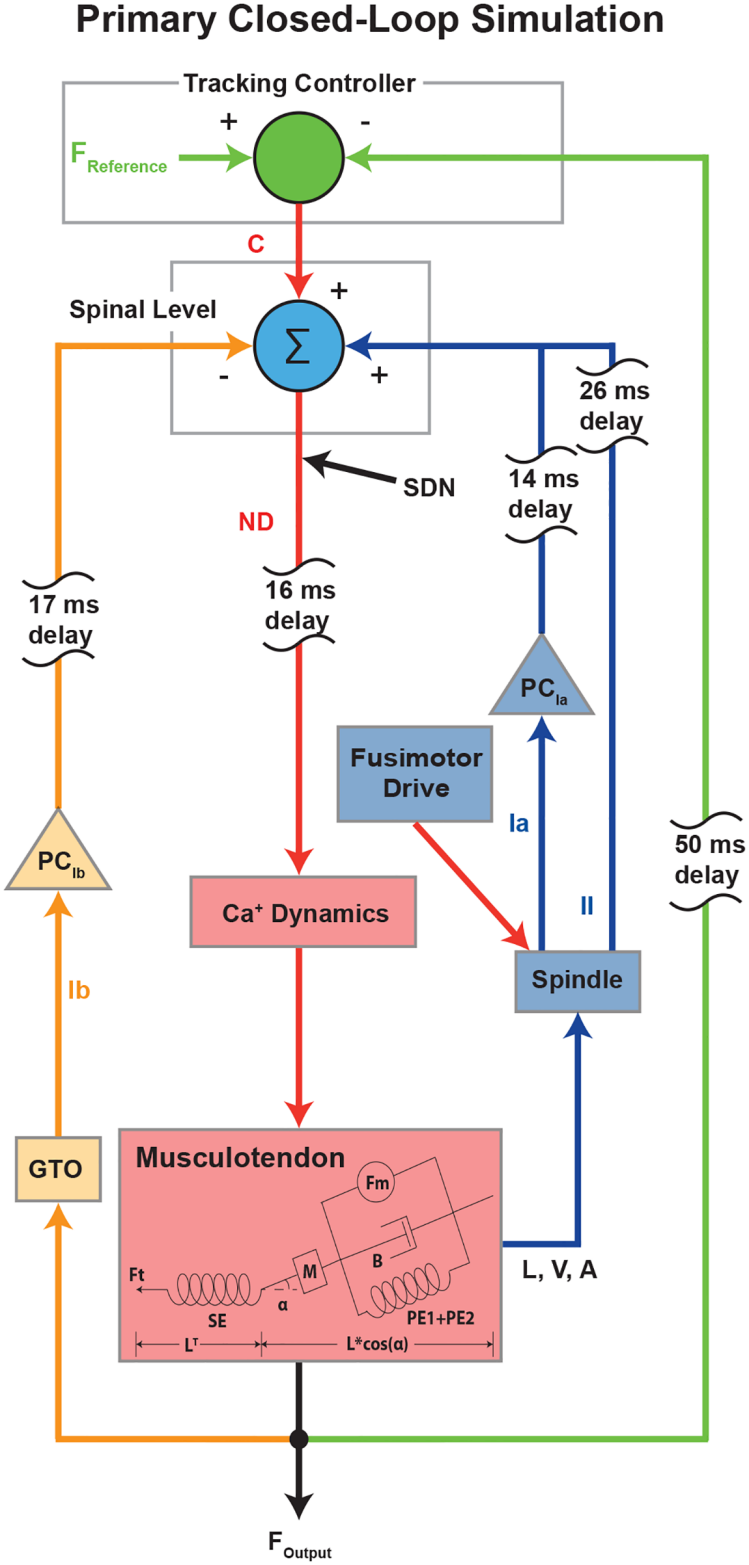
Primary closed-loop simulations of affrented muscle model. A musculotendon unit receives a neural drive (ND) derived from three input sources and signal dependent noise (SDN). The muscle spindle generates excitatory inputs through group Ia and II afferents at a given fusimotor drive level. The Golgi tendon organ (GTO) sends an inhibitory input through the group Ib afferent pathway. The input contributions of Ia and Ib afferents are controlled by presynaptic control (PC). The tracking controller provides a control signal (C) based on a fraction of the error between reference force (F_Reference_) and actual force output (F_Output_). These inputs are integrated at the spinal level and signal dependent noise (SDN) is added. The resulting neural drive (ND), filtered to account for Ca^+^ dynamics, induces contraction of the muscle (length (L), velocity (V), acceleration (A)), taking into account mechanical factors such as the pennation angle (*α*), mass (M), viscosity (B), parallel (PE1 and PE2) and series elastic elements (SE). Each afferent or efferent pathway has associated delays which account for the conduction velocities of each fiber, the distance between the spinal cord and the muscle of 0.8 m [34] and synaptic delays of 2 ms [35].

**Fig 2:**
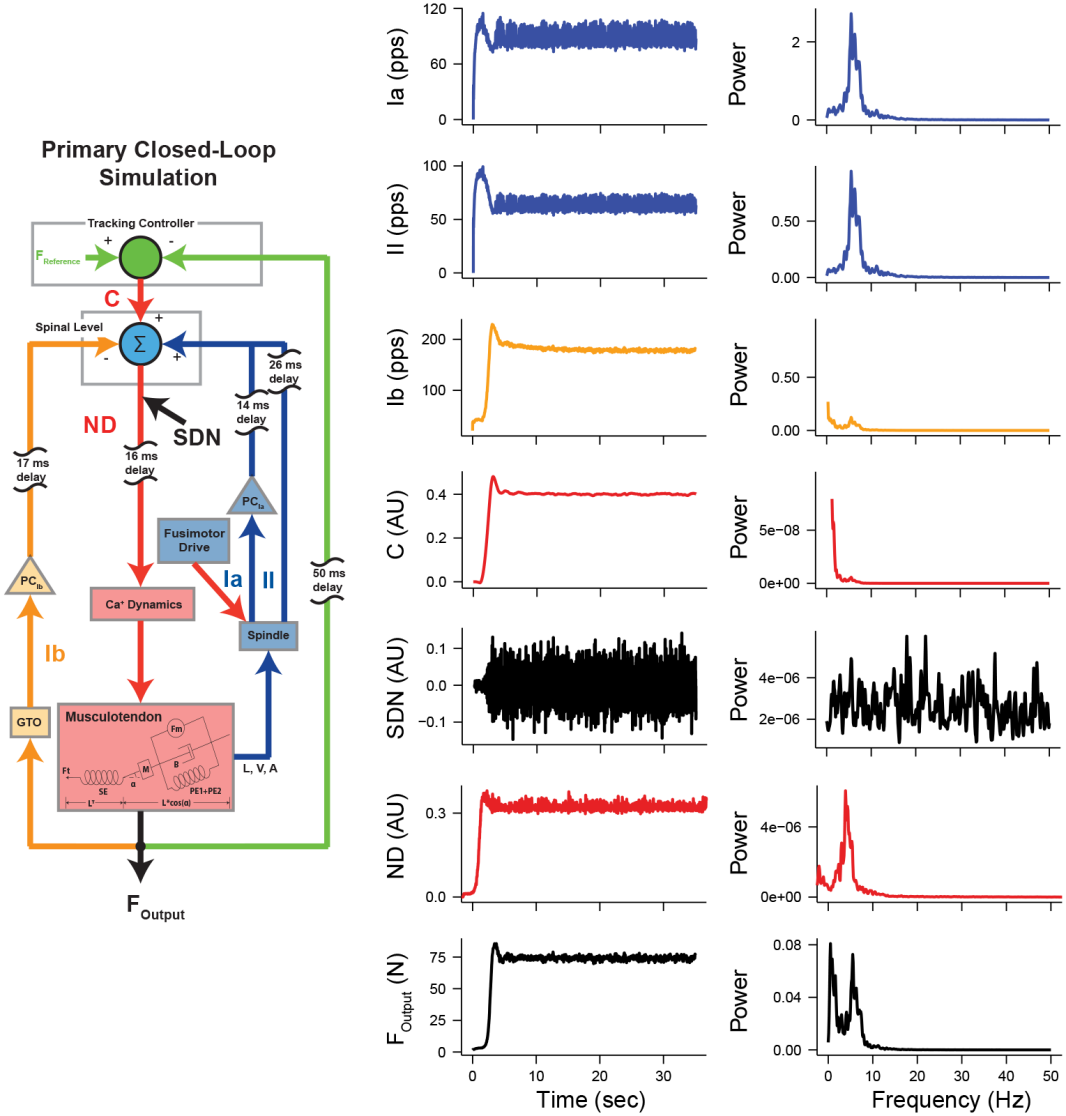
Example time-series signals and their power spectra obtained from the primary closed-loop simulation of an afferented muscle.

We used a closed-loop simulation of an afferented muscle model, which is an extension of a previously published model [28] (Details are given in Materials and methods). With this model, we identified the sources of frequency-specific force variability, and characterized interactions among them. We simulated an isometric force tracking task at 20% of maximal voluntary contraction (MVC). The schematic diagram of the model is provided in Fig 1. The afferented muscle model is comprised of a musculotendon unit [29–32], muscle spindle [33], and Golgi tendon organ (GTO) [34], which is driven by a tracking controller to ensure successful force tracking tasks [28]. Fig 2 shows sample time-series signals of each element from the closed-loop simulation of the afferented muscle model, along with their respective power spectra.

**Fig 3:**
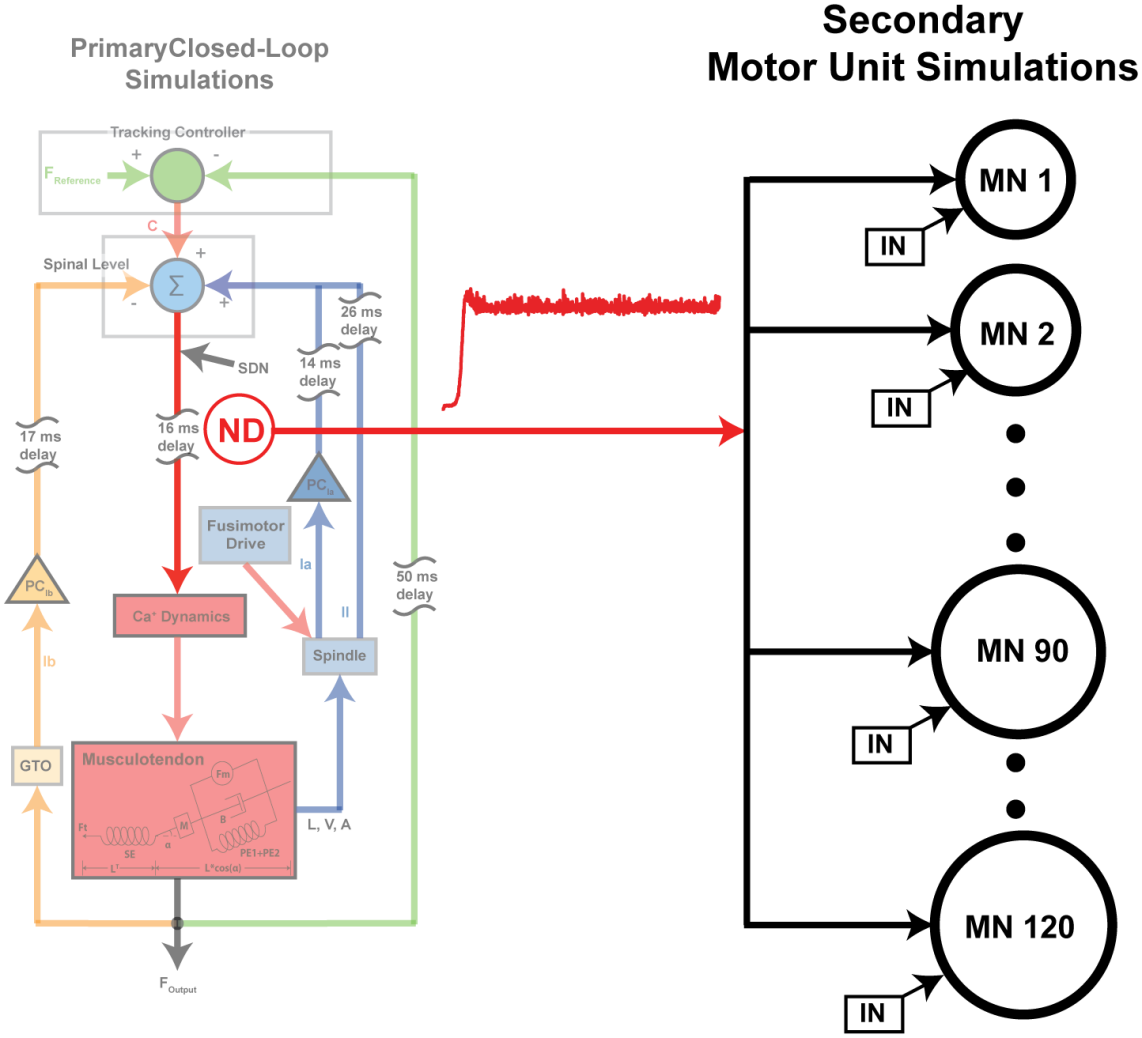
Secondary motor unit simulations. We used the neural drive (ND) obtained from the closed-loop simulations of an afferented muscle to investigate changes in synchronization patterns of motor units in different simulated conditions. The simulated neural drive is fed to a simulated motor unit pool as a common input across the pool. The motor unit pool consists of 120 motor units. Individual motor units also receive independent noise (IN).

We also performed sets of secondary simulations to investigate whether or not changes in involuntary force variability characterized in the closed-loop simulations can be detected as synchronization of motor unit activities within a pool. We considered this to be an important link to the literature because experimental studies consider synchronization across motor units as a measure of ‘effective’ (i.e., force-generating) neural drive to muscle [14]. Accordingly, synchronization across motor units has been used extensively to characterize the neural drive that generates involuntary force variability [25–27, 36–38]. In this set of secondary simulations, we took the neural drive generated by the primary closed-loop simulations (Fig 3) and fed it into a simulated pool of 120 motor units [39] as a common input. The resulting total discharge rate variability arising from independent as well as common synaptic input ranged from 17–33% of coefficient of variation, which is compatible with that observed experimentally [40]. The synchronization between randomly chosen pairs of motor units was quantified in both time and frequency domain using (i) common drive index [36] and (ii) coherence [26, 27, 41].

### Simulation 1: Closed-loop control of musculotendon

First, we investigated the interactions between mechanical properties of the musculotendon and broad-band neural noise using an open-loop input without any feedback (Simulation 1.1). For this simulation, our control input was simply the target trajectory (i.e., 1-sec zero input, 2-sec ramp-up and 32-sec hold at 20% MVC), with added signal-dependent noise. The coefficient of variation of force was 8.73%.

**Fig 4:**
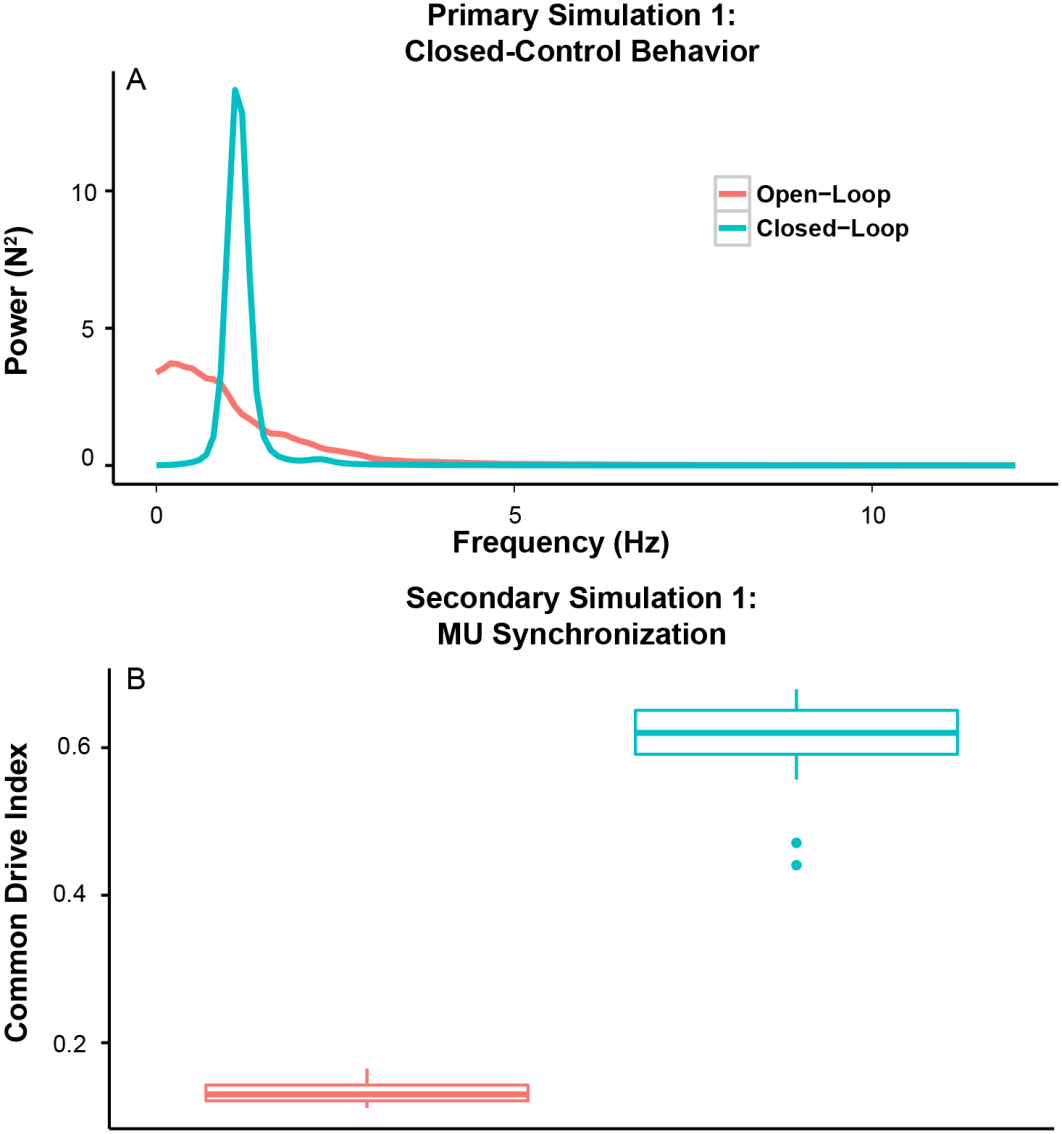
Low-frequency ‘common drive’ originates in musculotendon mechanics but is shaped and amplified by closed-loop control of muscle force. (A) Power spectra of output force from the afferented muscle model using two types of control strategies (open-loop control in red and closed-loop (error correction mechanism only) control in green). Noise level was adjusted to match the amplitudes of overall force variability between the two control strategies. (B) Motor unit synchronization in the common drive range (1-5 Hz) as per the common drive index for the two control strategies. Note that open-loop control in the presence of broad-band noise produces only low-frequency force fluctuations (below 5 Hz), which can be attributed to the low-pass filtering nature of musculotendon. Also, note that the closed-loop control only with a tracking controller results in generation of a distinct peak within the common drive range and increased common drive.

This open-loop control resulted in force variability which fell almost entirely below 5 Hz, within the ‘common drive’ range (red line in Fig 4A). It is worth noting that there was no distinct peak within this frequency range (i.e., 1-5 Hz). Accordingly, the neural drive produced in this simulation also caused a small degree of common drive, as measured by the ‘common drive index’ (red boxplot in Fig 4B). A similar result was observed using motor unit coherence analysis. It is also important to note that high-frequency force variability (5-12 Hz) did not arise from the interaction between mechanical properties of musculotendon and broad-band noise.

We then ran the simulation in closed-loop condition using only the error correction mechanism (i.e., tracking controller) (Simulation 1.2). The amplitude of overall force variability was 8.39%, which was not significantly different from the open-loop condition (independent sample t-test using Yuen’s method, p = 0.19). This addition of an operational tracking controller resulted in the generation of a peak at ∼ 1.8 Hz in the power spectrum of muscle force (green line in Fig 4A). Also, the degree of motor unit synchronization in this range increased accordingly (green boxplot in Fig 4B). These results altogether suggest that low-frequency force variability and common drive are primarily an emergent property of a close-loop control of muscle force. Also, these results show that high-frequency force variability does not emerge in the absence of proprioceptive feedback.

### Simulation 2: Ia afferent feedback

Gain control of Ia afferent feedback at the spinal cord, often experimentally quantified by H-reflex amplitude, plays an important role in human motor control and learning to achieve a variety of movements [42, 43]. Here, we examined how changes in the gain of Ia afferent feedback, modeled as presynaptic control input, influence force variability. We systematically altered the level of this presynaptic control input from the value of -0.5 to 0 while keeping the other gain parameters constant (70 pps for dynamic and static fusimotor drives and -0.3 for presynaptic control level of Ib afferent feedback). This range was set such that the mean input contribution of Ia afferent feedback to the neural drive spanned a range from 0 (i.e., no contribution from Ia afferent feedback) to 30% of the maximum neural drive.

**Fig 5:**
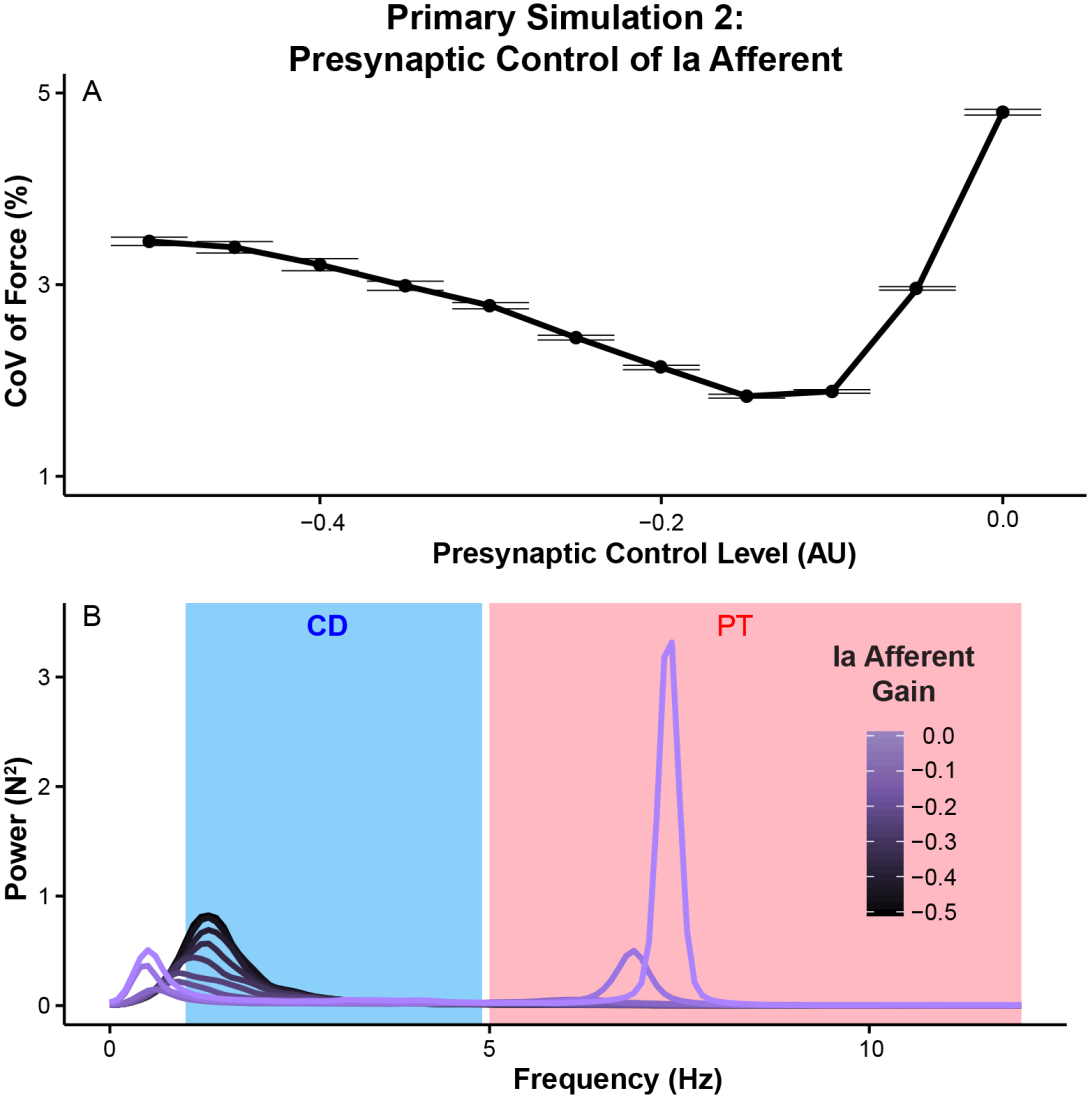
Modulation of Ia afferent feedback gain (i.e., presynaptic control input) influences the amplitude of overall force variability through its effects across the force-relevant frequencies. (A) Coefficient of variation (mean ± SE across 20 trials) at each presynaptic control level of Ia afferent feedback. The smaller the value of presynaptic control level, the lesser the contribution from this pathway. (B) Power spectra of force (mean across 20 trials at each level of presynaptic control). Power spectra were divided into two frequency ranges: 1-5 Hz common drive (CD) and 5-12 Hz physiological tremor (PT). Note that overall force variability shows U-shaped response to changes in the gain of Ia afferent feedback. Also, note that those changes in overall force variability occur across the range of frequencies below 12 Hz with distinctive peaks in the common drive and physiological tremor ranges.

The amplitude of force variability decreases as the presynaptic input level is increased and becomes minimal at the value of -0.15 (Fig 5A). Further increases negatively affect the amplitude of force variability (the presynaptic control level of -0.05 and 0 in Fig 5A). Analyses of force variability in the frequency domain show the change in force variability amplitude occurred across the frequency range (p < 0.01 at all the frequencies between 1 and 12 Hz), but prominent peaks exist in the two distinct frequency ranges, namely the common drive range (1-5 Hz) and physiological tremor range (5-12 Hz) (shown as blue and red bands for common drive range and physiological tremor range, respectively, in Fig 5B). These observations demonstrate that modulation of the strength of Ia afferent feedback is an important factor that influences overall force variability during ‘isometric’ force production.

**Fig 6:**
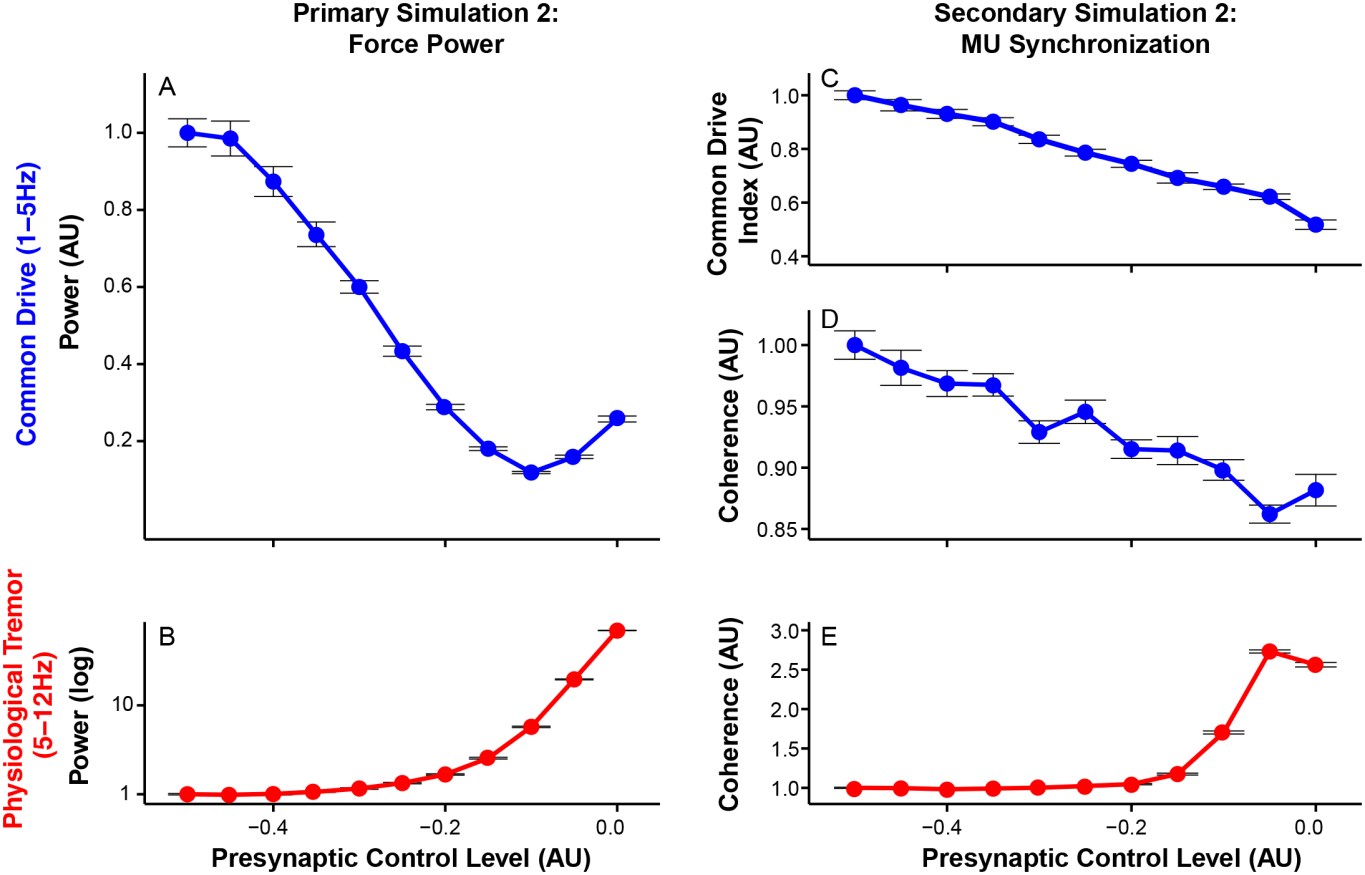
Increases in the gain of Ia afferent feedback (i.e., larger presynaptic control level) decrease low-frequency force variability (1-5 Hz) while they increase physiological tremor (5-12 Hz). All of the dependent variables are normalized to their respective mean values at the presynaptic control level of -0.5. (A) Mean force power within the common drive range (mean ± SE across 20 trials). (B) Mean force power within the physiological tremor range (mean ± SE across 20 trials). (C) Common drive index (CDI, mean ± SE of mean CDIs across 20 trials) (D) Coherence (mean ± SE of mean Fisher z-transformed coherence across 20 trials) in the common drive range. (E) Coherence in the physiological tremor range (mean ± SE of mean Fisher z-transformed coherence across 20 trials). Note that increases in the gain of in Ia afferent feedback (i.e., decreases in presynaptic inhibition of Ia afferent feedback) result in decreases in low-frequency force variability and increases in physiological tremor. Also, note that changes in motor unit synchronization generally reflect changes in force variability.

Further analyses on frequency-specific effects of Ia afferent feedback show increasing the gain of Ia afferent feedback reduces force variability within the common drive range (Fig 6A) while it increases the amplitude of physiological tremor (Fig 6B). Excessive Ia gain led to excessive physiological tremor as suggested in previous studies [20, 44]. As Ia afferent feedback increases, common drive decreases more than physiological tremor increases, after which physiological tremor dominates the spectrum and a monotonic increase in total force variability is observed. These observations suggest that the U shaped response comes from the relative contribution of common drive and physiological tremor to total force variability. Importantly, these concurrent changes in the common drive and physiological tremor are consistent with previous speculations [25–27]. These observations suggest that relatively faster excitation cycles of Ia afferent feedback can function as a negative feedback (i.e., withdrawal of Ia afferent input during muscle shortening and its excitation during muscle stretch), thereby interrupting the development of low-frequency force fluctuations, characteristic of a close-loop control of muscle force.

Changes in motor unit synchronization in the common drive and physiological tremor ranges are shown in (Fig 6C–6E). Stronger Ia afferent feedback reduces the degree of common drive (Fig 6C and 6D). In contrast, it induces a higher degree of synchronization in the physiological tremor range (Fig 6E). These results further confirm a previously suggested relationship between the strength of Ia afferent feedback and motor unit synchronization in the common drive and physiological tremor ranges [25–27].

#### Additional simulation 2.1: Contribution of tracking controller to reduction in low-frequency force variability

To determine the specific mechanism through which increases in the strength of Ia afferent feedback reduce low-frequency force variability, we ran two additional sets of simulations. The first (Simulation 2.1) aimed to address the question of whether changing the relative contribution of the tracking controller with respect to the total input could have resulted in the reduction in low-frequency force variability. We injected an additional constant excitatory input (amplitude of 1.7% of the maximum neural drive, equivalent to the Ia contribution when the presynaptic control value is -0.1), while the presynaptic control level of Ia afferent feedback was held at -0.5 to remove its contribution. All the other gain parameters were same as in Simulation 2. This additional constant excitatory input, however, did not change force variability in the common drive range (independent sample t-test using Yuen’s method, p = 0.27), nor the degree of motor unit synchronization (p = 0.87), compared to the original condition where there was no additional excitatory input (presynaptic control level of -0.5 in Fig 3). This result suggests that the reductions in low-frequency force variability cannot be explained by reduction in the relative strength of the tracking controller alone.

#### Additional simulation 2.2: Frequency response of closed-loop system

**Fig 7:**
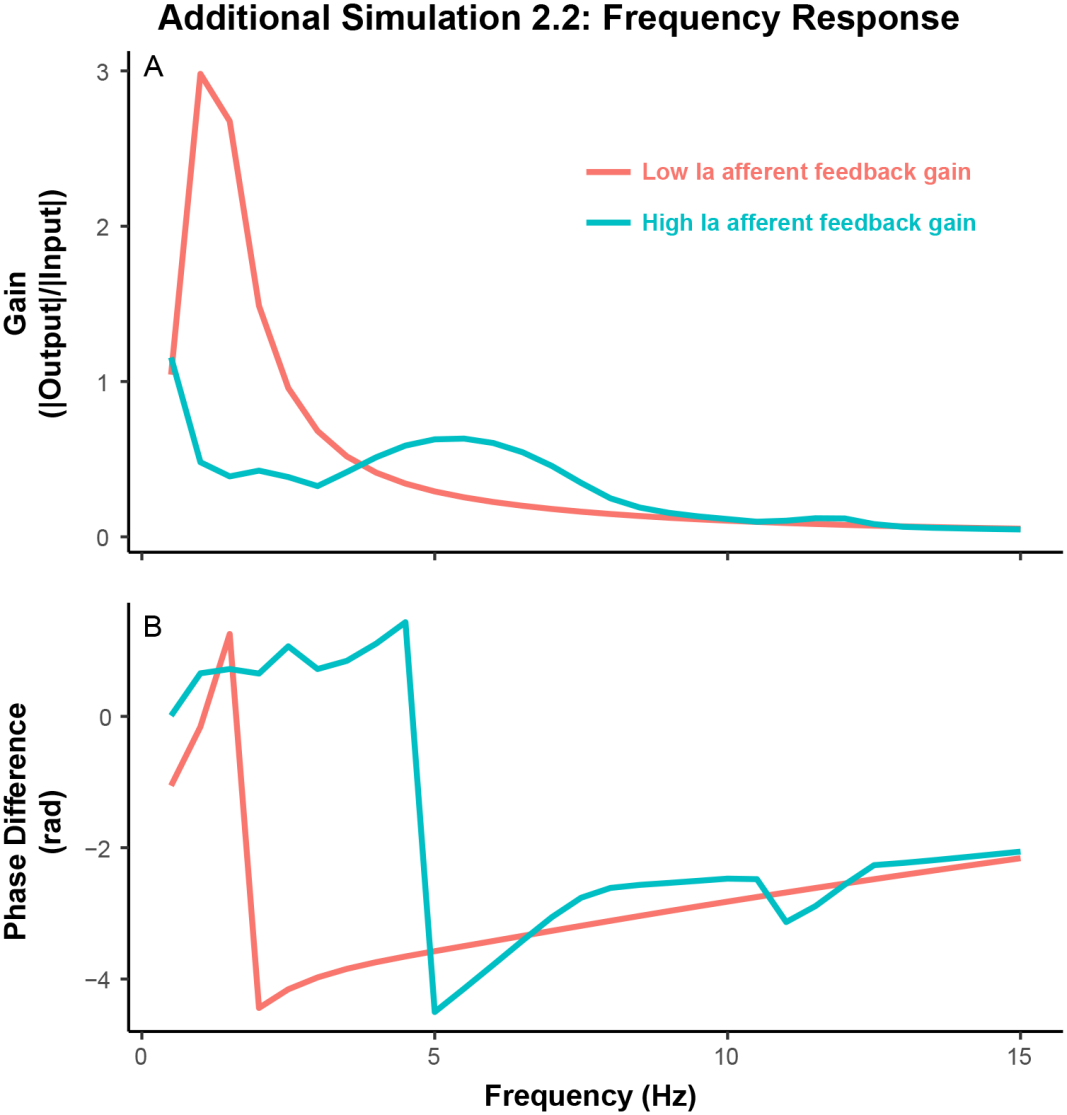
Frequency response of the closed-loop afferented muscle system with or without Ia afferent feedback. (A) gain of the system quantified as the ratio of the amplitude of output force, normalized to MVC, to the amplitude of input sinusoid. (B) phase delay of output force with respect to input sinusoids. Note that addition of Ia afferent feedback attenuates the amplification of low-frequency inputs and removes phase delays, present in the absence of Ia afferent feedback.

In the second set of simulations (Simulation 2.2), we quantified the frequency response of the closed-loop afferented muscle by removing the signal dependent noise and adding sinusoidal inputs of different frequencies with equal amplitudes. For each input, we quantified the gain (i.e., ratio of output amplitude to input amplitude) and phase delay (difference in phase between input and output). The Bode plots in Fig 7 characterize the consequences of altering presynaptic control on the frequency response of the system. Fig 7 shows that increasing Ia afferent feedback attenuates low-frequency inputs and removes associated delays seen in the absence of Ia afferent feedback. These results show that Ia afferent feedback induces a high-pass filtering behavior on the system, which prevents the development of slow force fluctuations and enables more rapid corrections. This is a previously unrecognized functional contribution of muscle afferentation.

### Simulation 3: Fusimotor drive

Understanding the operation of the fusimotor system is hindered by the lack of techniques which can directly measure *γ*-motoneuron activities [45]. However, experimental evidence based on human group Ia and II afferent activities has suggested that humans have control over the fusimotor system which is independent of *α*-motoneuron drive, and which can be modulated by attention and task requirements [46–48]. Here, we postulate that fusimotor-induced changes in the dynamic sensitivity and static bias of Ia afferent activity will have profound effects on force variability as well. Therefore, we tested three scenarios; 1) co-modulation of *γ* dynamic and static fusimotor drives, 2) modulation of *γ* dynamic or 3) *γ* static fusimotor drive independently while the other is held constant, as done previously [33]. In this study, we varied them from 10 to 250 pps by increment of 20 pps. When *γ* dynamic or static fusimotor drive was varied independently, the other was kept at 70 pps. The presynaptic control levels of Ia and Ib afferent feedback were set at -0.15 and -0.3.

**Fig 8:**
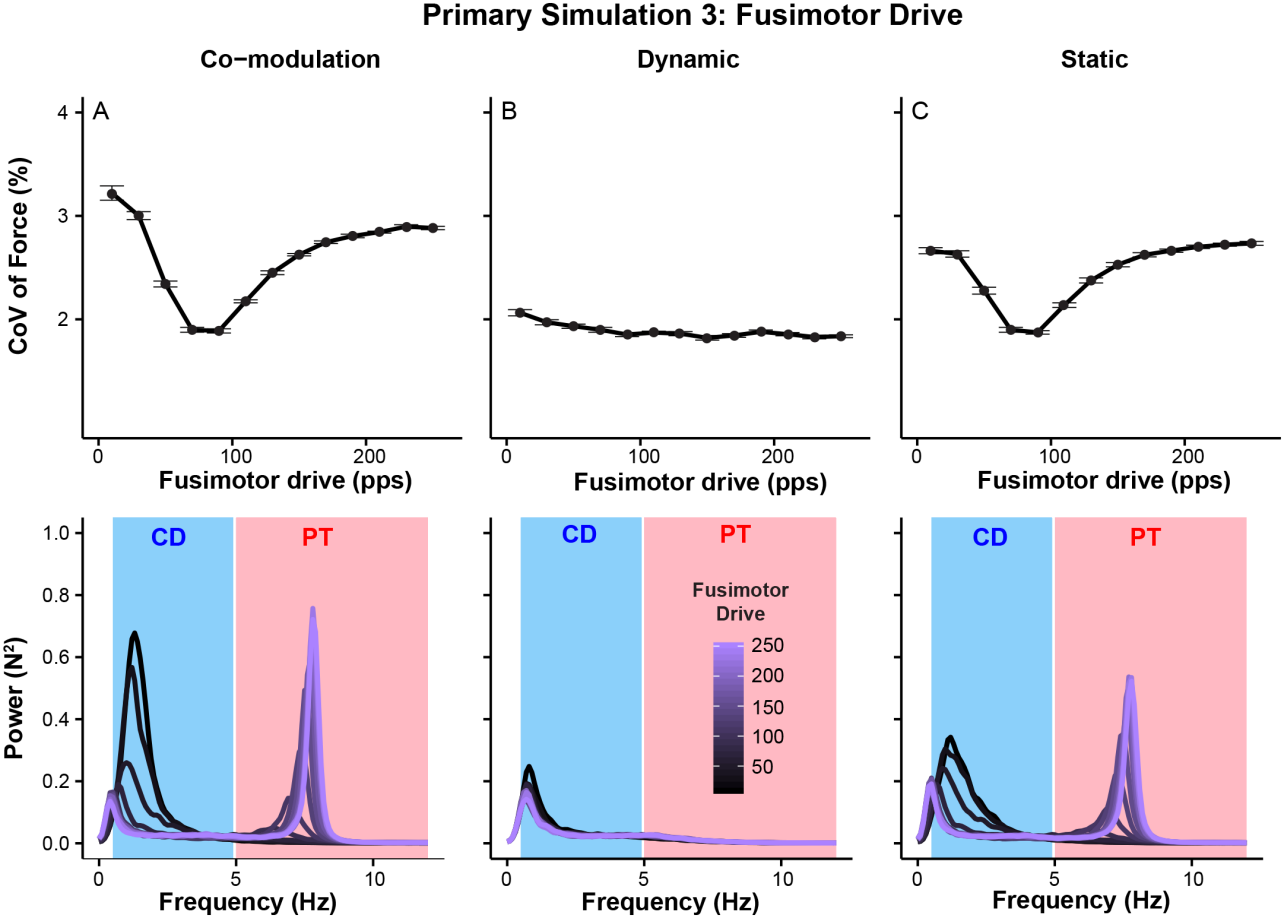
Modulation of Ia afferent activity through the fusimotor system affects the amplitude of force variability through its effects across the force-relevant frequencies. The top figure panel depicts the coefficient of variation of force and the bottom figure panel shows power spectra of force. (A) Dynamic and static fusimotor drives were co-modulated. (B) *γ* dynamic fusimotor drive was varied while *γ* static fusimotor drive was kept constant at 70 pps. (C) *γ* static fusimotor drive was varied while *γ* dynamic fusimotor drive was kept constant at 70 pps. Note that overall amplitude of force variability is less sensitive to modulation of *γ* dynamic fusimotor drive, while that of *γ* static fusimotor drive has significant effects on the amplitude of overall force variability. Also, effects of concurrent increases in *γ* dynamic and static fusimotor drives are a combination of their respective contributions. Importantly, those changes in overall force variability predominantly occur in the common drive range and physiological tremor ranges.

Results show that the amplitude of overall force variability depends on the levels of fusimotor drives (Fig 8A–8C top figures). When both *γ* dynamic and *γ* static fusimotor drives are varied, the amplitude of overall force variability shows a similar response to the presynaptic manipulation of Ia afferent feedback (Fig 8A top figure). Also, the changes again occur predominantly in the common drive and physiological tremor ranges (p < 0.01 at all the frequencies between 1 and 12 Hz) as indicated by prominent peaks in those ranges (Fig 8A bottom figure). Independent modulation of the only *γ* dynamic fusimotor drive has comparably smaller effects on the amplitude of overall force variability (Fig 8B top figure). On the contrary, modulation of *γ* static fusimotor drive produces effects similar to co-modulation of both fusimotor drives (Fig 8C top figure). Again, their effects occur in the common drive and physiological tremor ranges (Fig 8B and 8C bottom figures). These results show that the fusimotor system, especially *γ* static fusimotor drive, has profound effects on force variability in a frequency specific manner similar to presynaptic modulation of Ia afferent gain. This differential sensitivity to *γ* dynamic and static fusimotor drives might speak to differences in their functional significance during isometric force production. Also, it is important to note that too high levels of *γ* static fusimotor drives can lead to greater overall force variability accompanied by excessive physiological tremor, which might be similar to effects of fatigue [7, 49] (see Discussion).

**Fig 9:**
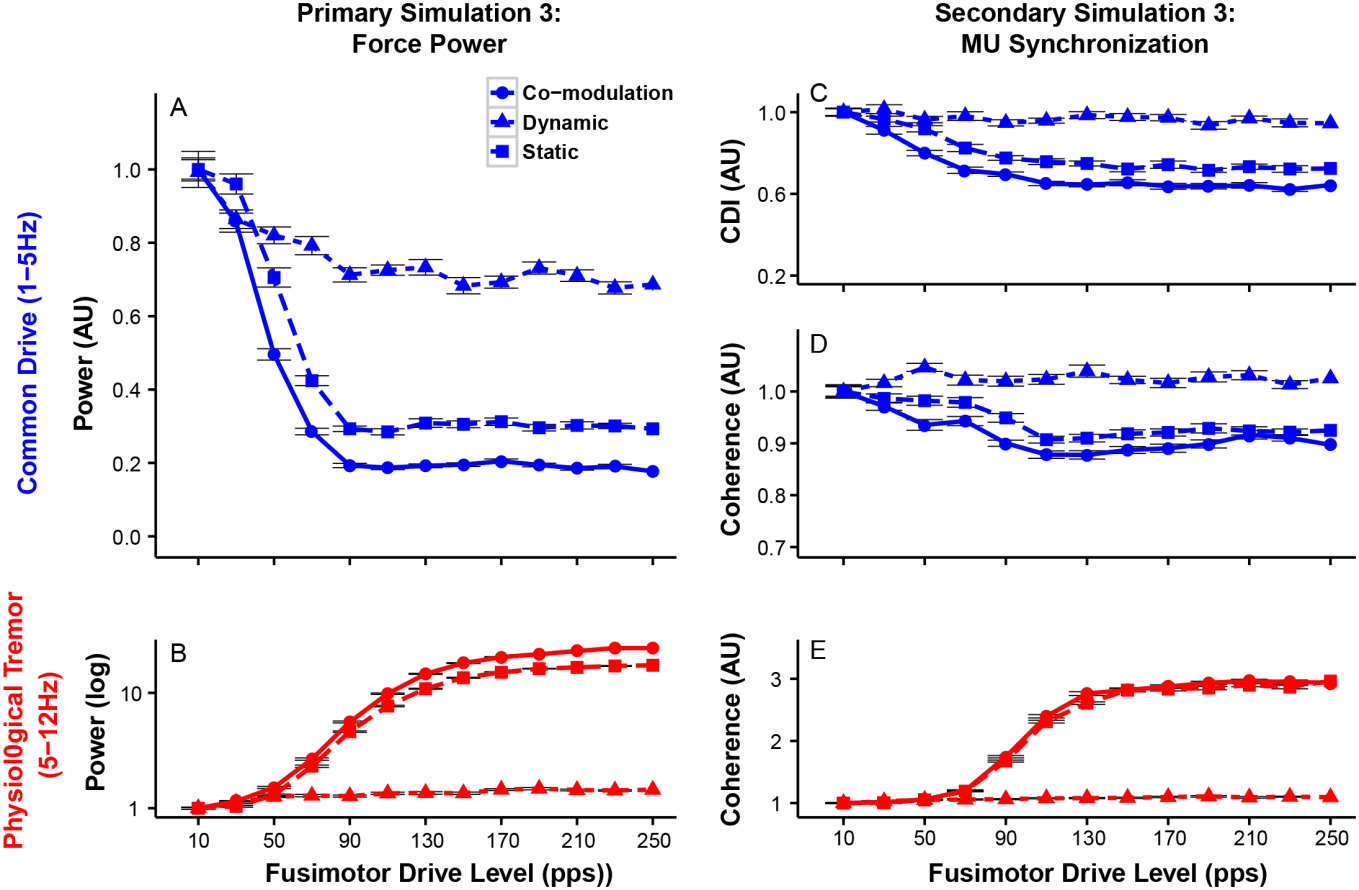
Modulation of Ia afferent activity through the fusimotor system decreases low-frequency force variability (1-5 Hz) while it increases physiological tremor (5-12 Hz). Three combinations of fusimotor drive modulation are indicated as follows; co-modulation of *γ* dynamic and static fusimotor drives (circles with solid line) and modulation of *γ* dynamic (triangles with dashed line) or *γ* static (squares with long-dashed line) fusimotor drives alone. All of the dependent variables presented here are normalized to their respective mean values at the lowest fusimotor drive for each condition. (A) Mean force power within the common drive range (mean±SE across trials). (B) Mean force power within the physiological tremor range (mean±SE across 20 trials). (C) Common drive index (CDI, mean±SE across 20 trials). (D) Coherence (mean±SE across 20 trials) in the common drive range. (E) Coherence (mean±SE across 20 trials) in the physiological tremor range. Note that changes in force variability and motor unit synchronization in both frequency ranges can be attributed primarily to effects of *γ* static fusimotor drive.

Further analyses in the two frequency ranges show greater fusimotor drives are associated with smaller force variability in the common drive range and larger physiological tremor (Fig 9A and 9B). The effects of *γ* static fusimotor drive are substantially larger than those of dynamic fusimotor drive in both frequency ranges and the combination of those effects is illustrated in the case of co-modulation of *γ* dynamic and static fusimotor drives. These results are consistent with those from presynaptic Ia afferent feedback gain such that increased bias level (mean input contribution) of Ia afferent feedback, rather than the dynamic sensitivity of Ia afferent feedback, plays a more important role in shaping the power spectrum of force variability and generating physiological tremor [50].

Changes in motor unit synchronization correspond well to changes in force variability, as shown in Fig 9C–9E. Greater fusimotor drives result in lower CDI values and low-frequency coherence (Fig 9C and 9D), as well as higher coherence in the physiological tremor range. These results suggest that modulation of *γ* dynamic and static fusimotor drives can also alter the degree of motor unit synchronization across the force-relevant frequencies.

### Simulation 4: Ib afferent feedback

Given that Ib afferent feedback in general provides inhibition of *α*-motoneurons as a function of force level, one can easily expect that it helps stabilize force fluctuations [45, 51]. However, exactly how such a feedback system influences either overall amplitude or frequency-specific components of involuntary force variability is unknown. Here, the presynaptic control value of Ib afferent feedback was varied from -0.5 to 0, while the presynaptic control value of Ia afferent feedback was kept at -0.3 and dynamic and static fusimotor drives at 70 pps. This range corresponds to a Ib contribution of 0 to 45% of the maximum neural drive, respectively. The upper range of these values would be non-physiological as the Ib input contribution of 45% of the maximal neural drive, for example, means 45% total input is continuously inhibited and it requires other compensatory mechanisms through Ia afferent feedback and a tracking controller to maintain the target force level. Here, we merely try to fully characterize effects of Ib afferent feedback on force variability and thereby highlight differences between Ia and Ib afferent feedback.

**Fig 10:**
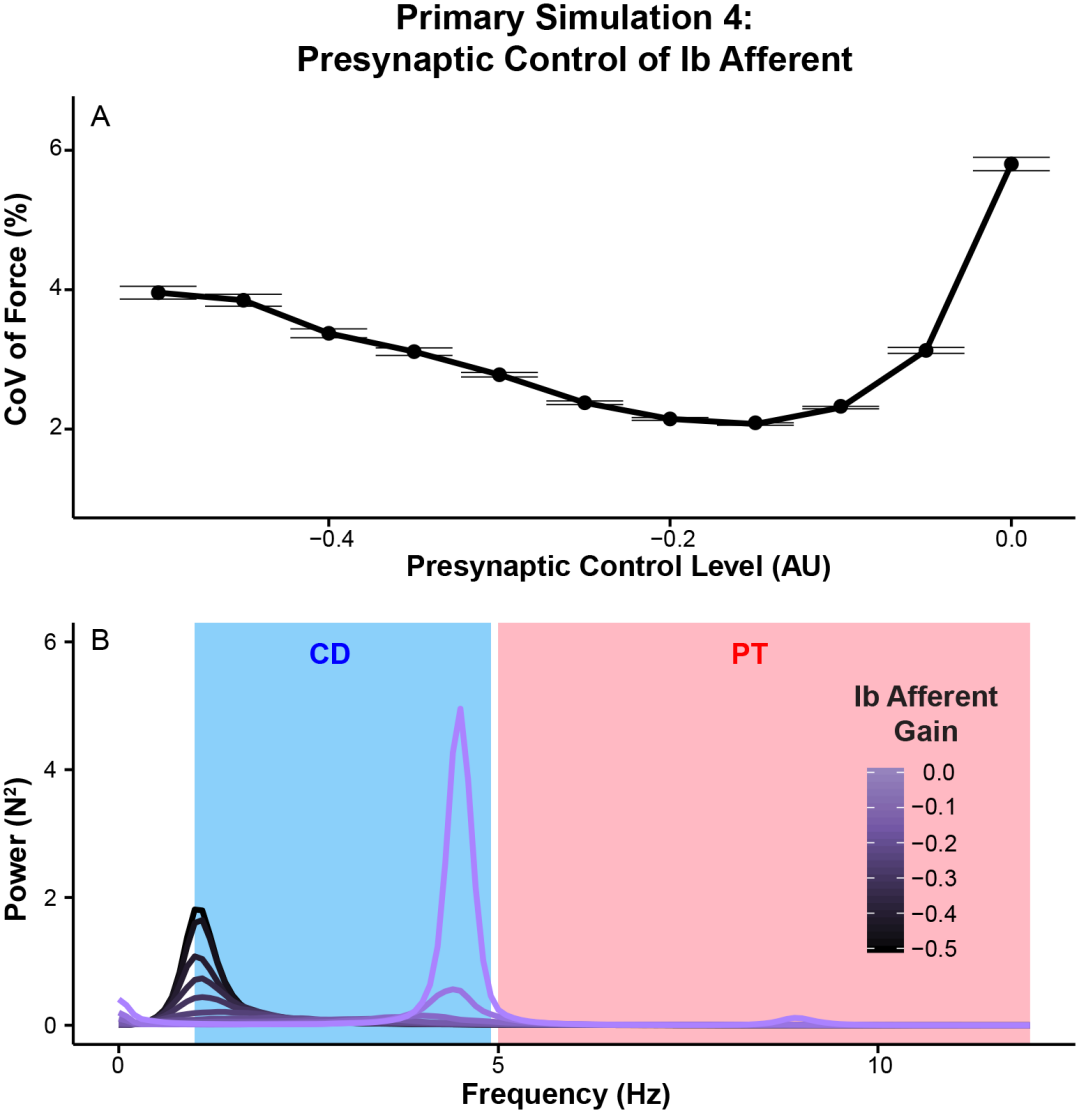
Modulation of presynaptic control of Ib feedback alters the amplitude of overall force variability mainly through its effects on the common drive range. (A) Ib-related changes in coefficient of variation (mean±SE across 20 trials). The smaller the value of presynaptic control, the smaller the contribution from the pathway. (B) Changes in power spectra of (mean across 20 trials) as a function of presynaptic control levels of Ib afferent feedback. Note the U-shaped response of the amplitude of overall force variability with increases in the strength of Ib afferent feedback. Also note that effects of Ib afferent feedback occur mainly in the common drive range.

As expected, greater inhibition of *α*-motoneurons through Ib afferent feedback reduces the amplitude of overall force variability (Fig 10A). However, excessive Ib gain can also lead to increased force variability at ∼4 Hz (Fig 10A) although it requires non-physiologically large Ib input contributions. In the frequency domain, changes in force variability occur across the frequencies (p < 0.01 at all the frequencies between 1 and 12 Hz), but mainly in the common drive as indicated by peaks appearing only in that range (Fig 10B). The slightly lower frequencies at which the second peak occurs compared to those of Ia afferent feedback might result from the longer loop delay of Ib afferent feedback (Fig 10B). These results highlight that Ib afferent feedback can regulate force variability much like presynaptic/fusimotor modulation of Ia afferent feedback, but its effects are mostly confined in the common drive range.

**Fig 11:**
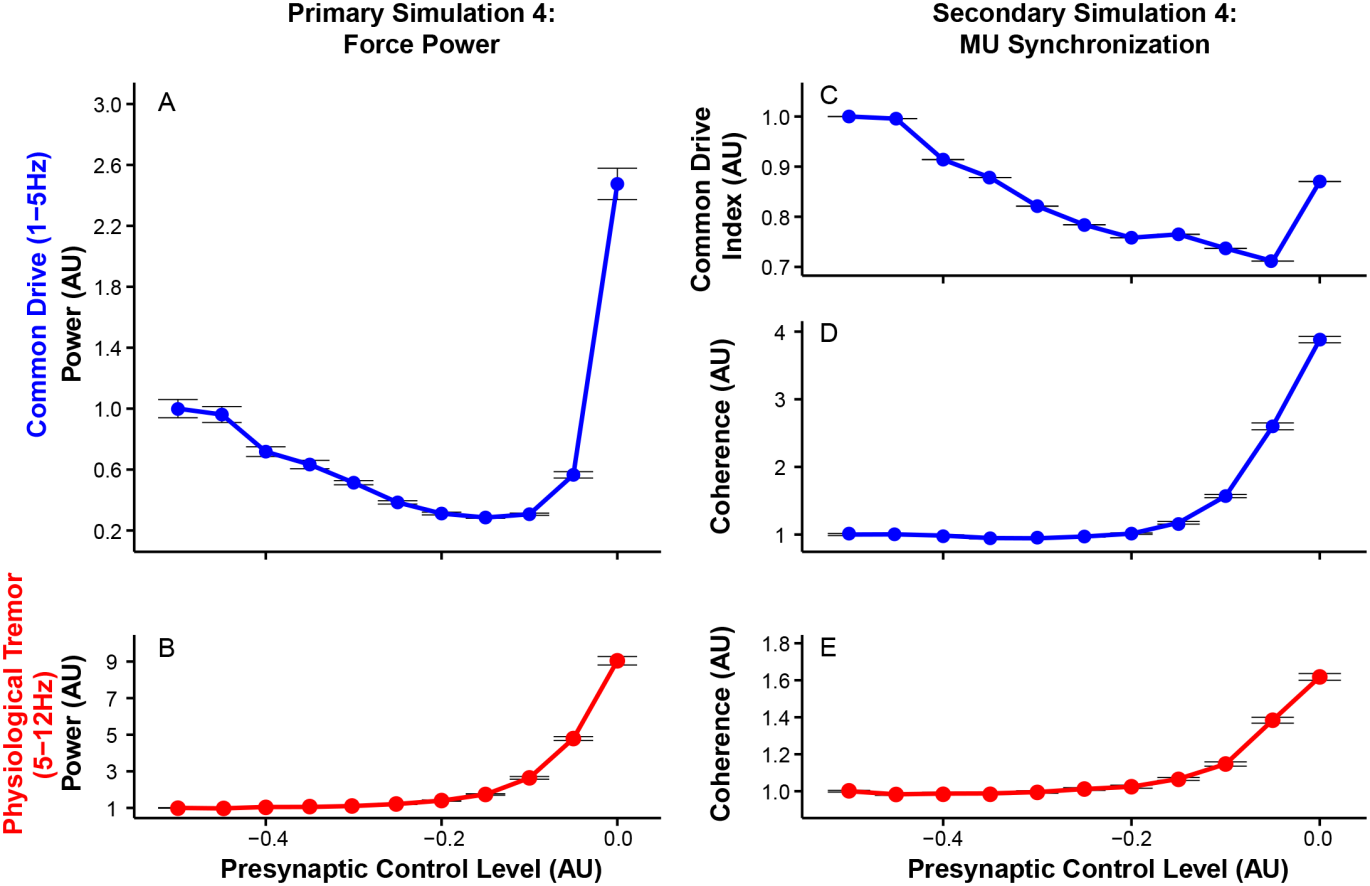
Increasing the strength of Ib afferent feedback decreases low-frequency force variability, but can lead to excessive force fluctuations in that range. All of the dependent variables are normalized to their respective mean values at the lowest presynaptic control level. (A) Mean force power within the common drive range (mean±SE across 20 trials). (B) Mean force power within the physiological tremor range (mean±SE across 20 trials). (C) Common drive index (CDI, mean±SE across 20 trials). (D) Coherence (mean±SE across across 20 trials) in the common drive range. (E) Coherence (mean±SE across 20 trials) in the physiological tremor range. Note that increased strength Ib afferent feedback preferentially affects force variability in the common drive range. Also note that CDI and coherence show different responses to excessive force fluctuations in the common drive range.

Increasing the strength of Ib inhibition results in smaller force variability in the common drive range (Fig 11A), but excessive Ib inhibition can lead to excessive force fluctuations in this range as shown in (Fig 10A). Its effects on physiological tremor are considerably smaller than presynaptic/fusimotor modulation of Ia afferent feedback (Fig 11B). These results highlight the differences in cross-frequency interactions between Ia and Ib afferent feedback pathways, which has not been reported previously.

As before, the frequency-specific effects of presynaptic Ib modulation on force variability are also reflected in motor unit synchronization (Fig 11C–11E). Higher Ib feedback gain is associated with lower synchronization in the common drive range and higher synchronization in the physiological tremor range. Interestingly, CDI and coherence in the common drive range respond differently to excessive force fluctuations at ∼4 Hz seen with excessive Ib inhibition (Fig 11C and 11D), suggesting that these two measures have differing sensitivity to synchronization at different frequencies within 1-5 Hz.

## Discussion

A series of closed-loop simulations of an afferented muscle show that many cardinal features of involuntary force variability emerge from closed-loop neuromechanical interactions. Our results reveal that closed-loop control of a viscoelastic musculotendon unit, combined with the tuning of proprioceptive feedback gains, naturally generate both low-frequency (1-5 Hz) force variability and high-frequency oscillations analogous to physiological tremor (5-12 Hz). Moreover, we show that these low- and high-frequency phenomena are in fact mechanistically related to each other—which suggests novel and fruitful directions for future research. This study is, to our knowledge, the first to directly confirm mechanistic links between low- and high-frequency force variability, as was proposed earlier [25–27]. Finally, we also used the emergent time histories of closed-loop net neural drive (‘ND’ in Fig 1) to drive the model of a motor unit pool. We find that these inputs suffice to produce motor-unit synchronization compatible with experimental findings [25–27].

Involuntary force variability at low frequencies (1-5 Hz) can arise from various sources, including low-frequency variability in the neural drive to muscle (the so-called ‘common drive’) [36]. As such, the amplitude of this common drive is a contributor to error during voluntary control of precision forces [14, 15, 36, 52]. Although common drive has been studied for over 30 years, its origins remain debatable [10].

Our results are significant because they suggest that common drive can emerge due to a combination of factors inherent to any neuromuscular control loop. Foremost among them is the viscoelasticity of the musculotendon, which acts as a mechanical low-pass filter that naturally allows the preferential conversion of low frequencies in the neural drive into muscle force as previously shown in [53–55]. It is this low frequency component (1-5 Hz) of muscle force that would be selectively reinforced by any imperfect physiological error correction mechanism. Thus, our results demonstrate that low-frequency force variability emerges naturally when controlling viscoelastic muscles—and do not require the presence of proprioceptive feedback. This is a novel alternative to other peripheral explanations. For example, Watanabe and Kohn suggested that high-frequency neural drive can be demodulated into lower frequencies [18], which still remains to be tested. In fact, our results are congruent with previous evidence for peripheral mechanisms, such as the fact that common drive persists even after disruption of the cortico-spinal tract, as in capsular stroke [41].

Another component of force variability is oscillations in the 5-12 Hz range, often called ‘physiological tremor.’ Physiological tremor may arise from multiple factors [56]. One of the earliest and most well-supported mechanisms is cycles of excitation around the stretch reflex loop [19, 20, 22]. The first important implication of our results is that, in contrast to common drive, physiological tremor does require the proprioceptive feedback in order to arise as shown experimentally [19, 22] and in computational simulations [20]. Thus mechanical resonance of musculotendons as proposed by [23, 24], did not suffice. In fact, we could not elicit physiological tremor via interactions between broad-band noise and the mechanical properties of musculotendon using an open-loop input which consisted of the target trajectory and signal-dependent noise. This result is consistent with previous experimental evidence and simulation [19, 20, 22].

Moreover, our simulations allowed us to characterize how physiological tremor amplitude is modulated by proprioceptive pathway gains. Those include both presynaptic control levels of inhibition/disinhibition (‘PC_*Ia*_’ & ‘PC_*Ib*_’ in Fig 1) and ‘descending’ *γ* fusimotor drive to muscle spindles (‘fusimotor drive’ in Fig 1). This detailed characterization was not possible in the previous simulation study by Stein and Oguztoreli [20] and added a new insight that physiological tremor amplitude is mostly determined by the bias level (i.e., mean input contribution) of Ia afferent feedback, not dynamic sensitivity of muscle spindle. Importantly, excessive Ia afferent gains could produce excessive oscillations *primarily* in the physiological tremor range in Figs 5 and 8, similar to what has been shown previously in animal models [44]. Interestingly, excessive Ib afferent gains could lead to excessive oscillations in the lower frequency range (3-5 Hz) possibly due to the longer delay along this pathway (Fig 10). These findings are particularly important to design hypotheses about how peripheral mechanisms interact with descending neural drive to produce physiological and other kinds of tremor in healthy and pathological conditions [16, 17, 57, 58].

Although we find that proprioceptive feedback is not strictly necessary to generate common drive, we do find that it can influence its strength. This is compatible with experimental findings [25–27]. Specifically, De Luca and colleagues report a negative correlation between the degree of common drive and muscle spindle density [25]. Further, Laine and colleagues showed that heightening the perception of task-related errors during a force tracking task led to increases in physiological tremor and H-reflex—while common drive decreased [26, 27]. Their interpretation was that the changes in common drive and physiological tremor both stemmed from the tuning of proprioceptive gains due to alterations in psycho-sensory state [46–48, 59–61]. These lines of experimental evidence, however, could not test a mechanistic link between common drive and physiological tremor. Here, we show that increasing the strength of proprioceptive feedback (via ‘PC_*Ia*_’ and ‘*γ* static fusimotor drive’) increases physiological tremor but concurrently decreases common drive (Fig 6). Thus, our results demonstrate that peripheral mechanisms suffice to reproduce those experimental findings.

This close link between the amplitude of involuntary force variability and proprioceptive pathway gains (in Figs 5, 8 and 10) may explain many experimental findings. For example, removing proprioceptive feedback leads to greater overall involuntary force variability (i.e., smaller values of ‘PC_*Ia*_’, ‘PC_*Ib*_’ and ‘*γ* static fusimotor drive’ in Figs 5, 8 and 10). This is similar to what has been seen in patients with deafferentation [12].

Moreover, we show that excessive proprioceptive pathway gains result in greater overall force variability and excessive physiological tremor (i.e., larger values of ‘PC_*Ia*_’, ‘PC_*Ib*_’ and ‘*γ* static fusimotor drive’ in Figs 5, 8 and 10). Interestingly, fatigue can produce similar effects on force variability and physiological tremor [7, 8]; however, a precise mechanism for this phenomenon has not been established. The enhancement of physiological tremor in fatigue can be attenuated by blocking Ia afferent feedback [7]. Further, the sensitivity of stretch/tonic vibration reflex responses is enhanced during fatigue [49]. An emerging picture is that Ia afferent feedback gains are increased during fatigue, but it is not clear how this occurs (i.e., via presynaptic inhibition or fusimotor modulation), and it is not clear why fatigue influences overall force variability rather than just physiological tremor.

Biro and colleagues suggested that augmented Ia afferent feedback during fatigue reflects a fusimotor-dependent compensation for reduced descending drive [49]. This suggestion was based on previous findings in cat where 1) the activity of fusimotor system is enhance by activation of group III and IV afferents [62, 63], which respond to an accumulation of metabolites during fatigue [64, 65], 2) Ia afferent firing rates increase accordingly during fatigue contractions [62, 66], and 3) group III and IV afferents, on the contrary, enhance presynaptic inhibition of Ia afferent feedback [67]. Since presynaptic inhibition would reduce Ia afferent feedback gain, only the increased fusimotor activation seems a plausible compensatory mechanism.

Thus it is important to mention that, when we tested the effects of increased fusimotor drive in our simulation, the results of *γ* static fusimotor drive (‘*γ* static fusimotor drive’ in Fig 8) accurately predicted changes in force variability, as might occur during fatigue. Our findings therefore may provide a mechanistic link between several complementary lines of investigation related to fatigue.

As demonstrated in the cases of deafferentation and fatigue, the close link between our results and experimental findings may represent an important step in developing a unifying theory of human sensorimotor control that further relates spinal circuitry to manifestations of altered involuntary force variability under various neuromuscular conditions such as aging [1, 68, 69], stroke [10], cerebral palsy [9], Parkinson’s disease [13], and essential tremor [70]. For example, we show that increased Ia afferent feedback gains result in increased force variability below 0.5 Hz (i.e., larger values of ‘PC_*Ia*_’ and co-modulation and ‘*γ* static fusimotor drive’ in Figs 5 and 8). This might provide a link between increased force variability below 0.5 Hz seen in patients post stroke [10, 15] and their heightened Ia afferent feedback gains [71] or lower reflex threshold [72].

Thus, a unifying principle emerges. Namely, that the task-specific tuning of proprioceptive pathway gains in spinal circuitry—or its disruption—produces characteristic changes in the spectra of neural drive. Importantly, these can be quantified by measuring force variability.

Our results highlight the significance of considering closed-loop control of afferented muscle in the generation and modulation of involuntary force variability in motor control research. Historically, the force fluctuations have been considered as manifestation of ‘neural noise’ that is intrinsic to neural drive [73]. Despite the fact that such noise (e.g., signal dependent noise) is usually not frequency-specific, involuntary force fluctuations tend to be highly structured [14].

Our results now show that neuromechanical interactions impose structure onto noisy neural drive, and thus involuntary force variability and ‘noise’ are not independent, as is often assumed [15]. This idea may be significant in formulation of theoretical frameworks in motor control. For example, the ability of the proprioceptive feedback system to regulate the amplitude of overall involuntary force variability provides a neural mechanism to minimize it, as suggested by some [3, 74].

It is important to discuss how the limitations of our model do not affect our conclusions. Our afferented muscle model was not intended to represent the full complexity of the spinal cord circuitry. We used a simplified version of a previously described model of a spinal-like regulator [75, 76] that can replicate experimental behavior. Specifically, we did not include Renshaw inhibitory interneurons, which are known to provide recurrent inhibition of *α*-motoneurons and inhibition of Ia inhibitory internuerons [77]. However, in our simulation of a single muscle, the role of Renshaw inhibitory interneurons would be restricted to recurrent inhibition and therefore have effects similar to that of Ib inhibitory feedback, which we did include.

Secondly, our model did not attempt to replicate the exact biophysical structure of *α*-motoneurons and sensory afferents. Rather, we used a single-input/single-output structure to describe the population behavior of each system. We believe this simplification is reasonable because 1) the population response of an *α*-motoneuron pool is linear with respect to its common/shared synaptic input, since noise and non-linear properties of individual neurons get canceled out in the overall population behavior [14, 78], and 2) the common input to an *α*-motoneuron pool is the ‘effective’ neural drive, that is, the input that is actually translated into muscle force [14]. Therefore, it was appropriate for the contractile element in the afferented muscle to be modeled as a single input-output element. Another outcome of using a lumped parameter model of muscle is that force is not generated by the summation of twitches from progressively recruited motor units. However, neither physiological tremor nor ‘common drive’ is thought to relate directly to this aspect of physiological force generation [79]. It is also worth noting that since we simulated constant-force contractions, the number of units recruited/derecruited during each trial would have been very small and therefore would have only minor influence on the overall amplitude of force variability. Similarly, the population behavior of muscle spindles can be appropriately modeled as a single element, as muscle spindles are in general believed to distribute their synaptic inputs widely across a motor unit pool [80]. While potential non-uniformity of Ia projections has been suggested [81], this remains to be validated, and confirmed across different muscles.

Thirdly, we did not include modulation of *α*-motoneuron excitability through various neuromodulatory inputs arising from the brainstem, which can influence reflex sensitivity [82]. Such neuromodulatory effects would be widespread and more difficult to interpret, while also greatly increasing the complexity of our analyses.

Finally, our simulation was limited to that of a single muscle during isometric contraction, which is a valuable and informative experimental paradigm [1, 9, 26–28]. As in those experimental studies, it is difficult to extrapolate our findings to complex actions involving movement and coordination among multiple muscles. Still, we believe that our results help establish a strong basis for future study of peripheral and neuromechanical factors influencing the control of muscle force.

Lastly, we demonstrate that the modulation of involuntary force variability via proprioceptive pathway gains gives the nervous system a certain degree of control over involuntary force variability. Properly regulating those gains is important if disruptive tremor is to be avoided [44]. Our ability to understand and modify these relationships will be instrumental to providing insights into the neural mechanisms and circuits associated with functional performance [1], aging [1, 6], fatigue [7, 8] and neurological disease [9–13]. Finally, our approach of combining experimental observations with a computational simulation should provide a springboard for future investigation of neuromechanical interactions and task-dependent tuning of sensorimotor integration and proprioceptive mechanisms during voluntary actions in healthy development and aging; and disease.

## Materials and methods

### Closed-loop simulation of afferented muscle model

We used a closed-loop simulation of an afferented muscle model, which is an extension of a previously published model [28], to identify the sources of frequency-specific force variability and to characterize interactions among them. The schematic diagram of this model is provided in Fig 1. The afferented muscle model is comprised of a musculotendon unit [29–32], muscle spindle [33], and Golgi tendon organ (GTO) [34], which is controlled by a tracking controller [28]. The model was implemented in the MATLAB environment (The MathWorks Inc., Natick). Full details of each model are given in the corresponding references and only brief descriptions are provided here. All model parameters were taken from the corresponding references except for musculotendon architecture as described below.

#### Musculotendon model

The musculotendon model incorporates realistic physiological properties of muscle force production and resulting contraction dynamics [30–32]. Those properties include non-linear properties of passive elements of muscle and tendon, force-length and force-velocity relationships of contractile elements, and activation-force dynamics such as calcium kinetics, sag, yield, and activation-frequency relationship [83, 84]. Architectural parameters of the musculotendon model was adjusted according to those for the *tibialis anterior* muscle (Table 1) [34, 85]. The *tibialis anterior* was chosen because it is a large superficial muscle often chosen for motor unit analysis [25, 27].

**Table 1:** Model parameters for muscle based on architectural parameters of *tibialis anterior* muscle.

Mass (g)	150
Optimal fiber length (cm)	6.8
Tendon length (cm)	27.5
Pennation angle (deg)	9.6
Ia afferent conduction velocity (m/s)	64.5
II afferent conduction velocity (m/s)	32.5
Ib afferent conduction velocity (m/s)	59
*α*-motoneuron conduction velocity (m/s)	56
Synaptic delay (ms)	2

#### Closed-loop control system

A closed-loop controller enabled the entire system to perform a force tracking task at a constant force level as previously described [28]. The control signal (C) from a tracking controller and proprioceptive feedback signals were delayed and integrated at the spinal level (the box in Fig 1) to generate the neural drive (ND) to the muscle. The neural drive was then passed through a sigmoid transfer function, which describes the non-linear input-output behavior of a pool of motoneurons [75, 76]. In this study, signal dependent noise (SDN) was added to the neural drive such that the magnitude of output force variability closely matched experimental literature (∼ 2-10% coefficient of variation) [69]. The SDN was modeled as zero-mean, white noise signal low-pass filtered at 100 Hz (4th order Butterworth filter) with variance of 0.3. The sum of ND and SDN was delayed by 16ms to account for conduction delay along *α*-motoneuron and passed through the activation filter, which accounts for first-order calcium dynamics of muscle [31]. The resulting activation signal (MA) induces contraction of muscle and associated dynamics within the musculotendon. Delays along each afferent or efferent pathway accounted for the conduction velocities of each fiber, the distance between the spinal cord and the muscle of 0.8 m [34] and synaptic delays of 2 ms [35] (Table 1).

*Tracking controller:* The tracking controller was implemented to ensure successful force tracking at a given contraction level as in [28]. It continuously adjusts the level of the control signal (C) based on a fraction of the error between actual force output (F_Output_) and reference force (F_Reference_) as shown in Fig 1 and in the equation below,

$$\begin{array}{c} C=G\times (F_{Reference}-F_{Output}) \end{array}$$ (1)

where G refers to the gain of the tracking controller, which was set at 0.00035. The gain was empirically determined such that coefficient of variation of force is around the upper end of experimentally observed range (∼10%) in the absence of proprioceptive feedback.

This controller is not designed to reflect a specific neural circuit, but rather it is meant to describe general error correction mechanisms used during various motor tasks as done in our previous study [28]. Accordingly, the latency (total feedback loop delay of 66-ms) was set to approximately match the fastest EMG responses measured during tracking tasks [86], which is thought to involve the supraspinal circuits [87].

*Muscle spindle:* The muscle spindle model was adopted from [33]. This model generates Ia and II outputs as functions of muscle fiber length (L), velocity (V) and acceleration (A) through three types of intrafusal fibers (i.e., bag_1_, bag_2_, and chain fibers). Ia and II pathways provide monosynaptic and dysynaptic excitation of *α*-motoneuron, respectively. Also, each of intrafusal fiber receives fiber-type-specific fusimotor drive (dynamic and static) through *γ*-motoneuron.

*Golgi tendon organ:* The Golgi tendon organ (GTO) model was adopted from [34]. This model describes population behavior of GTOs and resulting Ib afferent activity. The GTO model converts tendon force into Ib fiber output using the transfer function described in [34]. The Ib afferent feedback was modeled as a disynaptic inhibitory pathway to *α*-motoneuron [34]. Although some evidence suggests convergence of Ia afferent feedback onto Ib interneurons [43] and reversal to positive feedback loops [88], we simplified the model as done previously [28, 34, 75, 76].

*Gain control of proprioceptive feedback:* We used gain control mechanisms of proprioceptive feedback pathways similar to ones used in the previous literature [75, 76]. Activity of individual afferents (Ia, II, and Ib) were first normalized to a value between 0 and 1. Each proprioceptive feedback pathway except for II afferents received presynaptic control input (PC_Ia_ and PC_Ib_ in Fig 1), whose value could range from -1 to 1. The sum of the normalized afferent activity and presynaptic control input was then passed through a sigmoid transfer function, which bounds the output to be a value between 0 and 1, to account for nonlinearity in the input-output relationship of a given cell population as described previously [75, 76]. This presynaptic control mechanism is a salient feature of gain control of propriocetive feedback systems at the spinal cord level, which can be controlled by various descending pathways as well as interneuronal circuits within the spinal cord [44, 89–91]. We did not consider presynaptic control of group II afferent feedback in this study because the connectivity of group II interneurons is not well understood [45]. However, we included weak excitatory input from II afferent feedback (∼ 2% of total input), as their weak contributions to stretch reflex have been suggested [77].

Another gain control mechanism for Ia afferent feedback is fusimotor drive through *γ*-motoneurons. *γ* dynamic and static fusimotor drives (fusimotor drive in Fig 1) alter responses of intrafusal fibers to muscle stretch and therefore the behavior of Ia and II afferent feedback from muscle spindles [33]. Dynamic fusimotor drive determines the dynamic response of muscle spindles, while static fusimotor drive determines the baseline level of Ia afferent activity and negatively affects the dynamic response [33]. Therefore, when dynamic and static fusimotor drives are concurrently increased, Ia afferents increase their static bias while their dynamic sensitivity is maintained [33].

### Primary simulation protocols

We simulated a force tracking task using a closed-loop simulation of the afferented muscle model. Each trial consisted of 1-s zero input phase, 2-s ramp-up and 32-s hold at 20% MVC. The last 30 s of each trial were used for further analysis. We simulated 20 trials for each condition described below.

In the first set of simulations (Simulation 1), we examined the frequency spectrum of output force variability arising from interactions among musculotendon, broad-band neural noise, and error correction mechanism. First, we simulated the force tracking task without the tracking controller and proprioceptive feedbacks (i.e., open-loop control) to characterize the interaction between mechanical properties of musculotendon and broad-band neural noise (Simulation 1.1). In this open-loop control condition, the neural drive consisted of open-loop input (i.e., 1-sec zero input, 2-sec ramp-up and 32-sec hold at 20% MVC) and signal dependent noise. The amplitudes of the open-loop input and noise were adjusted such that the mean force level was at 20% MVC and coefficient of variation of force equaled to that from the closed-loop condition described below. We made these adjustments so that 1) the same force/input level is used in both conditions, thus facilitating comparisons of force variability and motor unit synchronization, and 2) because normalizing the total force variability across conditions makes comparison of their spectral characteristics more straightforward.

Then, we replaced the open-loop input with the tracking controller (i.e., closed-loop control) to investigate how an error correction mechanism interacts with force variability arising from mechanical properties of musculotendon (Simulation 1.2). In this closed-loop control condition, proprioceptive feedback was removed by setting presynaptic control inputs to the value of -0.5 for each pathway.

In Simulation 2-4, we examined effects of proprioceptive feedback on force variability. To do so, we ran a set of simulations varying one of the proprioceptive pathway gains (i.e., presynaptic control level of Ia afferent feedback (Simulation 2), dynamic and static fusimotor drives (Simulation 3), and presynaptic control level of Ib afferent feedback (Simulation 4)) from its minimum to maximum values, while keeping the other gains constant. The minimum value corresponded to the value at which the contribution of that particular proprioceptive feedback is completely removed. The maximal value was determined empirically by the presence of non-physiological high-frequency force variability. The minimum and maximum values of fusimotor drive were set at 10 and 250 pulse per second (pps), respectively. At each parameter value, we run 20 trials.

Following Simulation 2, we ran two additional sets of simulations to investigate the mechanism through which increases in Ia afferent feedback gain lead to reductions in low-frequency force variability. In the first set of simulations (Simulation 2.1), we tested whether reductions in the relative strength of the tracking controller in response to increased excitatory input from Ia afferent feedback could explain that observation. To do so, we ran a set of simulations (20 trials) where the presynaptic control level of Ia afferent feedback was set at -0.5 and we added a constant excitatory input whose amplitude corresponded to the average input contribution from Ia afferent feedback at its presynaptic control level of -0.1. This presynaptic control value was chosen because we observed the smallest amplitude of low-frequency force variability. All the other gain parameters were held at 70 pps for dynamic and static fusimotor drives and -0.3 for presynaptic control level of Ib afferent feedback.

In the second set of simulations (Simulation 2.2), we quantified the frequency response of the closed-loop afferented muscle to investigate how addition of Ia afferent feedback changes the dynamics of this closed-loop system. To obtain the frequency response, we ran two sets of simulations using presynaptic control levels of Ia afferent of -0.5 and -0.1, while presynaptic control level of Ib afferent, dynamic and static fusimotor drives were kept constant at -0.3, 70, and 70, respectively. In these simulations, we removed the signal dependent noise and injected a set of sinusoids (0.5 to 15 Hz in steps of 0.5 Hz) with amplitude of 1% of the maximum neural drive 5 sec after the initiation of simulations. We quantified gain and phase of output force in response to an input sinusoid. The gain was computed as the ratio of the amplitude of output force to the amplitude of the input sinusoid. Phase was calculated as a phase difference between mean phase of the output force and input sinusoid during each trial.

### Secondary motor unit simulations

We performed secondary simulations for all conditions tested (Simulations 1-4) to investigate whether or not changes in the involuntary force variability produced in the closed-loop simulations can be detected as synchronization of motor unit activities within a pool. To do so, we used our simulated neural drive as a common input to a simulated motor unit pool obtained from [39]. While this model is simplistic by comparison with more biophysically-nuanced compartment-based models [34, 92, 93], it is nonetheless entirely sufficient to describe the basic phenomenon of motor unit entrainment by common input [94, 95] for the following reason. The ‘effective’ (i.e., force-generating) neural drive is common to all motor units within a pool [14]. Thus, the population activity is a linear transformation of the common input, even though individual motor unit responses to that input are nonlinear [96]. As a result, specific non-linearities present in each motor neuron response such as plateau potentials, adaptation, resonance, accommodation, etc., which can be modeled by those more complex models, are not an important consideration for our present application.

This motor unit model describes the orderly recruitment of motor units and rate coding in response to a common excitatory input. The motor unit pool consisted of 120 motoneurons, whose recruitment threshold to excitatory input had exponential distribution with a greater number of motoneurons with low thresholds and a small fraction of motoneurons with high thresholds as described previously [39]. The range of recruitment thresholds was set such that the highest value was 30-fold that of the lowest. Firing rate of a motoneurons was linearly scaled to excitatory input with a constant minimum firing rate of 8 imp/s. All of these properties were same as those in the original study [39]. We added discharge rate variability (5% CoV) to spike trains of individual motor units, indicated as IN in Fig 3, to simulate the effects of independent noise. In this simulation, we also included a previously modeled intrinsic property of motor unit known as persistent inward current [97].

### Data analysis

#### I. Force

The amplitude of overall force variability obtained from close-loop simulations of afferented muscle was quantified by coefficient of variation (CoV) of force. The force variability was further analyzed in the frequency domain. Power spectrum of the force was obtained using pwelch function in MATLAB with 5-s Gaussian window, 50% overlap, and the frequency resolution of 0.1Hz.

Statistical analysis was performed to identify dependence of force power at each frequency (dependent variable) on the level of proprioceptive feedback gains (independent variable). We used the heteroscedastic one-way ANOVA for means [98] from “WRS2” package in the R environment for statistical computing (The R Foundation for Statistical Computing, Vienna, Austria). Significance level was set to p < 0.05. Furthermore, force variability was analyzed in two frequency ranges (i.e., common drive (1-5 Hz) and physiological tremor (5-12 Hz)). Mean force power within each frequency range was calculated.

#### II. Motor unit synchronization

*Common drive index:* Common drive index (CDI) is a time-domain measure of synchronization between motor units in the 1-5 Hz range [36]. This value ranges from -1 to 1, 1 being perfect positive linear correlation and 0 being no correlation between motor units.

From the simulated motor unit pool, we randomly sampled 50 pairs of concurrently active motor units. Spike trains of each motor unit were smoothed with a 400-ms Hann window and high-pass filtered at 0.75Hz [36, 99, 100]. We calculated cross-correlations of pairs of the processed spike trains. CDI values were obtained by finding the peak correlation coefficient value within ± 100-ms time lag from the cross-correlogram [36, 100]. The correlation coefficient was transformed using Fisher’s z-transform (hyperbolic arctangent) before averaging [101]. For each iteration, the mean Fisher z-transformed CDI value across 50 pairs of motor units was calculated and transformed back to correlation coefficients using the inverse of the Fisher z-transformation [101]. This provided 20 CDI values for each condition.

*Coherence:* Similarly, synchronization between pairs of motor units was quantified in the frequency domain using coherence analysis [14, 26, 27, 102]. Coherence at each frequency ranges from 0 to 1, 1 being high synchronization at that frequency. Magnitude squared coherence between each randomly selected, unique pair of motor units (same as used in the common drive analysis) was calculated using 5-s Gaussian windows with 50% overlap and 0.1 Hz frequency resolution. Then, Fisher z-transformed coherence spectra were averaged across 50 pairs of motor units to obtain a single coherence spectrum for each condition. Mean Fisher z-transformed coherence values within the common drive (1-5 Hz) and physiological tremor (5-12) ranges were extracted from the averaged coherence spectrum for each trial.
